# Navigational Needs and Preferences of Hospital Patients and Visitors: What Prospects for Smart Technologies?

**DOI:** 10.3390/ijerph18030974

**Published:** 2021-01-22

**Authors:** Jan Ženka, Jan Macháček, Pavel Michna, Pavel Kořízek

**Affiliations:** 1 Department of Social Geography and Regional Development, Faculty of Science, University of Ostrava, Chittussiho 10, 710 00 Ostrava, Czech Republic; jan.zenka@osu.cz; 2 AGEL a. s., Jungmannova 28/17, Nové Město, 110 00 Praha 1, Czech Republic; pavel.michna@are.agel.cz; 3 AGEL Research and Training Institute, Mathonova 291/1, Krasice, 796 04 Prostějov, Czech Republic; pavel.korizek@msy.agel.cz

**Keywords:** wayfinding, hospital, spatial abilities, gender, age, smartphone navigation

## Abstract

In this paper, we map navigational needs and preferences of patients and visitors to evaluate the appropriateness of a smartphone navigation application in the hospital in contrast to other, more traditional navigational cues. We test the effects of sociodemographic variables (age, gender, education) on wayfinding strategies and preferences of respondents (using chi2 tests). Empirical research is based on the survey among 928 patients/visitors of the Vítkovice Hospital in Ostrava, Czechia. We found a relatively weak association between gender and wayfinding—no major differences between men and women in navigational preferences were found. Age was the most important predictor of wayfinding. Respondents in the over-60-year age group were characteristic of a lower interest in changes of the navigational system and low willingness to use mobile applications for navigation—people between 41 years and 60 years were the biggest supporters of changes. Correspondingly, demand for improvement of navigation (including a mobile application) was positively correlated with educational level.

## 1. Introduction

Wayfinding in large complex buildings, such as hospitals, has been an increasingly difficult task [1,2,3,4]. Navigational issues in hospitals cause stress and anxiety for the patients/visitors [1,2]. Correspondingly, they may lower the productivity of the staff members, who also face difficulties in wayfinding, and/or are frequently asked by the patients/visitors for help [5]. Previous research [6] showed that there is no universal solution: no navigational system is appropriate for all patients. Different groups of patients have different navigational needs [4]. Spatial cognition and wayfinding are affected by many factors, including the age [7], gender [8], education, health disabilities [9], and culture [10]. Wayfinding in large complex buildings is also highly contextual, depending on the particularities of urban design, architectural settings, and historical development of the place.

Probably the most progressive and promising solution of the wayfinding difficulties has been the utilization of navigation mobile applications based on indoor positioning. The development of a mobile application that will be useful for the patients/visitors/staff not only requires input from the big data analytics (see Gu et al. [11] for a conceptualization) capturing the prevailing route strategies, but also a survey of navigational needs among the (future) users. Patient participation might contribute significantly to improvements in healthcare services [12], including navigational systems. It is necessary to identify their current navigational strategies, and major barriers and obstacles in wayfinding and their willingness to participate in personal digitalization [13] for navigational purposes, which is the main aim of this paper. We also need to map a demographic structure of the respondents (age, gender, education) and consider their prevailing health disabilities, such as physical, visual, or cognitive impairment. Our primary research question is if, and to what extent, navigational preferences of the hospital patients/visitors are affected by sociodemographic factors: age, gender, and education.

This paper is based on a case study of the Vítkovice Hospital in the city of Ostrava, Czechia. Interviews with the hospital patients and visitors were conducted 928 times. Respondents were asked about their perception of the current hospital navigational system and preferences of various cues, focusing on their willingness to use a smart navigation application. We aim to contribute to the existing literature concerning wayfinding in hospitals by linking two different streams of research are linked: (i) theoretical studies focusing on explanation of age-, gender-, or education-based differences in wayfinding [8,14,15,16,17,18,19], and (ii) applied research dealing with the navigational systems in hospitals from the technology and/or management point of view [4,20,21]. Our aim is to provide specific recommendations for the design of hospital navigation systems that will be based on theoretical understanding and empirical identification of hospital patients’/visitors’ navigational needs.

In the following section, we provide a conceptualization of wayfinding and discuss the effects of selected sociodemographic determinants: age, gender, and education. The third section describes data and methods. In the fourth section, we present basic facts about the current navigational system in the Vítkovice Hospital. The fifth section provides basic empirical results, while the sixth section compares them with findings of other authors and discusses. In the seventh section we suggest solutions, and in the eighth, conclusions and implications for future research are drawn.

## 2. Wayfinding: Conceptualization and Determinants

For this text, we define wayfinding as “the process of finding your way to a destination in a familiar or unfamiliar setting using cues given by the environment” [22]. Wayfinding fundamentally depends on spatial orientation, defined as an ability to form a cognitive map [22]. A cognitive map can be understood as a mental construct of the experienced world used to understand and know the environment [23]. Successful spatial orientation depends on spatial abilities (such as the mental rotation, spatial perception, or object location memory), defined as “…the cognitive processes involved in locating targets in space, perceiving distance and directional relationships, and mentally transforming objects with respect to their position or orientation in space” (Lawton 2010, p. 318) [15]. Mental rotation, defined as “the ability to judge how an object would look if turned in two- or three-dimensional space” (Lawton 2010, p. 318) [15], is essential not only for mental construction of a congnitive map, but also for reading and using printed maps and recalling spatial information provided by those maps. Spatial perception (ability to identify verticality/horizontality) is used for a route learning based on vectors and Euclidean distances, while object location memory is important for a route learning based on landmarks [24]. Spatial orientation and spatial abilities are (among other factors) significantly associated with age, gender, and education.

### 2.1. Age

The ability of adults to find the way in space deteriorates with increasing age [25,26]. Authors dealing with the relationship between spatial orientation and age usually do not study the effects of age in isolation but focus on neurodegenerative diseases. Elderly people are often characterized by spatial orientation problems, especially when driving a car. Therefore, elderly people tend to avoid unfamiliar places and routes, which harms their mobility. They also have more problems remembering the route and determining the correct direction at orientation [27,28]. According to Newman and Kaszniak [29], seniors’ ability to store and recall information within spatial memory is impaired. Seniors also need more time to create a cognitive map of the surrounding environment and make more mistakes in navigation than young individuals [18]. Studies on gender differences suggest that men at different ages [30] generally score better than women in spatial orientation tests [31,32]. Moffat and Resnick [33] state that older individuals are guided more by adjacent landmarks when navigating the space and are unable to make adequate use of distant landmarks. This implies that their ability to make and use cognitive environment maps is limited. The results suggest that allocentric navigation strategies, in which cognitive maps are used, deteriorate in increasing age [16]. Correspondingly, Rodgers et al. [19] argue that the elderly rely dominantly on the egocentric navigational strategy, while younger individuals use both strategies roughly equally. Many older people have better self-orientation in a familiar environment [25], but are difficult to navigate in an unfamiliar environment. The problem grows with age [27]. Seniors are also generally more self-confident about their sense of direction than younger generations [34]. Borella et al. [35] found that age has a significant impact on spatial skills, but it depends on the spatial task. Despite this research, it is hard to make unambiguous conclusions about the impact of age on spatial orientation. Smart technologies and mobile applications could help solve the mentioned problems with orientation. However, there are several problems with the use of mobile apps by elderly people. For visually impaired users, it is difficult to read instructions or use a map on a small mobile device display [36]. The next problem is that some people may not have a smartphone or may not have one at the moment, although the share of people in the over-60-year age group (further only 60+) having access/using mobile Internet has been growing rapidly [37].

**Hypothesis 1.**  *People in the over-60-year age group differ from younger people in their navigational preferences*.

**Hypothesis 2.**  *People in the over-60-year age group are less willing to use smart technologies for wayfinding than younger people*.

### 2.2. Gender

There is plenty of empirical evidence supporting the thesis that men and women differ in their spatial abilities, spatial orientation, perception, and wayfinding strategies [8,15,38,39,40,41,42,43,44,45]. “Gender differences in spatial abilities are considered among the largest gender differences in all cognitive abilities” [41,46]. For this study, we identified three major gender differences that may significantly affect the navigational needs of men and women. Firstly, the majority of studies confirmed that men had better results in tests of (some) abstract spatial abilities, such as mental rotation, as well as in spatial orientation in the real environment [41,47]. Secondly, women generally showed higher levels of spatial anxiety and uncertainty than men [38,43,48]. Thirdly, men and women differed significantly in their wayfinding strategies [40,49].

Starting with the first point, according to the results of Lawton [8], men tend to be more efficient than women in mental rotation, more accurate in their judgments of directional relationships, locating hidden targets in real or simulated environments, pointing to out-of-sight landmarks, or “when answering questions about directional relationships of locations previously seen on a map” [8,50]. Women, on the other hand, can better memorize landmarks and their spatial distribution [8]. While many studies found no significant gender differences in navigational abilities and wayfinding (see Coluccia and Louse [41]), women are generally less confident about their ability to find a destination in the real environment, including buildings [51]. Lawton [38] argues that this stress reduces their ability to follow the cues necessary to maintain spatial orientation, and reduces memory for spatial locations. Therefore, we expect gender differences to significantly affect the navigational needs of the patients and visitors.

**Hypothesis 3.**  *There are gender differences (more women than men) in the opinion that the newcomers face difficulties in finding a destination in the hospital*.

**Hypothesis 4.**  *Women appreciate improvements in the navigational system of the hospital more than men*.

As already noted, men and women differ substantially in their navigational preferences and strategies for finding a destination [40]. Men tend to rely more on global reference points, such as the position of the sun in the sky [38,51], cardinal directions (north, west, etc.), and Euclidean distances (“turn after 200 metres”)—“the orientation strategy”. Women tend to focus on the left-right turns, follow a sequence of landmarks (“turn left near the pizzeria”), that is, the “route strategy”, and show better landmark memory—see Lawton [38], Dabbs et al. [52], or Liao and Dong [43]. Nevertheless, “…the fact that men tend to use global configurations and geometric information does not contradict with the fact that they can also employ landmark information for wayfinding” [43]. These differences occur not only in the wayfinding strategies, but also in giving directions.

Men are more prone to using allocentric strategies of wayfinding [38,51] that are independent of the navigator’s position, referring primarily to the spatial configuration of landmarks. Women, on the other hand, use an egocentric strategy, characteristic by a self-location based on cues such as turns, distances, and directions [40,48]. More importantly, there is empirical evidence that forming 3D representation based on 2D images is more difficult for women than for men [42]. However, there is no conclusive finding concerning gender differences in using maps for wayfinding [43]. Some authors found that men had better map reading skills [53,54]; see also Havelková and Hanus [55] (for review) and were more accurate in the wayfinding based on maps [56], whereas other authors found no significant differences [39,57]. Women were found to prefer written or verbal instructions when travelling to a new destination more than men [58,59].

Based on the above-mentioned differences in spatial abilities (mostly in mental rotation) and the wayfinding strategies, we expect the following relationships between gender and navigational needs of our respondents.

**Hypothesis 5.**  *Men and women differ in their navigational preferences*.

The traditional digital divide model [60] assumes that usage and adoption of the telecommunication technologies are associated with the sociodemographic variables: age, gender, and education. Adoption of technologies deteriorates with growing age and improves with increasing education. People with a higher level of formal education should adopt technologies faster than people with a lower level. Men are assumed to adopt technologies faster than women. This is, however, a valid assumption only for the early phase of the adoption of the technology. While there might be minor gender differences in the adoption of healthcare information systems (e.g., men participate more in online health communities, see Liu et al. [61]), we found no conclusive empirical evidence that men are more/less prone to use mobile (navigation) applications than women (see Hwang et al., 2016 [62]). Concerning the ability and willingness to use smart navigation technologies, Silber-Varod and Hacohen [17] argue that “the difference between men and women has diminished and even vanished over the first decade of the 21st century”. Therefore, we expect that:

**Hypothesis 6.**  *There are no gender differences in the willingness to use a mobile navigation application*.

### 2.3. Education

Education levels have an impact on an individual’s cognitive function [63,64], including spatial abilities. The effects of education on spatial abilities are usually not studied in isolation, but in interaction with age and gender. The wayfinding abilities develop gradually over a lifetime [65]. Wiederholt et al. [66] or Proust-Lima et al. [67] tested the effects of age, gender, and educational level on cognitive abilities of elderly people. Both papers indicate higher cognitive levels (including visual memory) of more educated respondents and gender differences in visuospatial skills favouring men. However, while Proust-Lima et al. [67] document steeper cognitive decline in the group of more educated people, Wiederholt et al. [66] argued that the rate of cognitive decline with increasing age was slower in the college-educated group. Ulrich et al. [16] found that gender, age, regional urbanization, and education proved to be relevant sociodemographic determinants for wayfinding. According to their results, higher orientation scores showed people who lived in smaller urban regions had higher levels of education, and more males and age 35+ respondents compared to those who were females, younger, and less educated, residing in large cities (more than 500,000 inhabitants). Educated people also showed higher scores in the route strategy. Behavioural research also points to a link between higher education and better spatial orientation [16,67]. A different view, given the nature of the participants in the research, is held by Růžičková et al. [68]. They targeted people with visual impairments, arguing that the orientation in the space of such people is not influenced by the level of education, but rather by an alternative way of accepting space in terms of behavioural geography.

**Hypothesis 7.**  *More educated people differ from people with lower education in their navigational preferences*.

## 3. Context: The Vítkovice Hospital and Its Current Navigational System

The Vítkovice Hospital was established in 1853 as the first company hospital (for the workers in the Vítkovice steel mill) in the Austria–Hungarian Empire, and is one of the oldest in Europe. The most rapid development of the hospital took place during the world wars and in the 1990s. From 1993 to 2000, the hospital belonged to the statutory city of Ostrava, and since 2000 the hospital has been owned by the private company AGEL a.s. The Vítkovice Hospital is currently a general hospital with an extensive inpatient and outpatient department, providing comprehensive care, especially in the area of Ostrava-Vítkovice and Zábřeh. The hospital has 848 employees (2019), 12 wards, and 25 outpatient clinics [69]. The number of hospitalizations in 2019 was 15,516, ambulatory number of examinations 240,000, and a unique number of outpatients was 97,000 [69].

**Figure A1:**
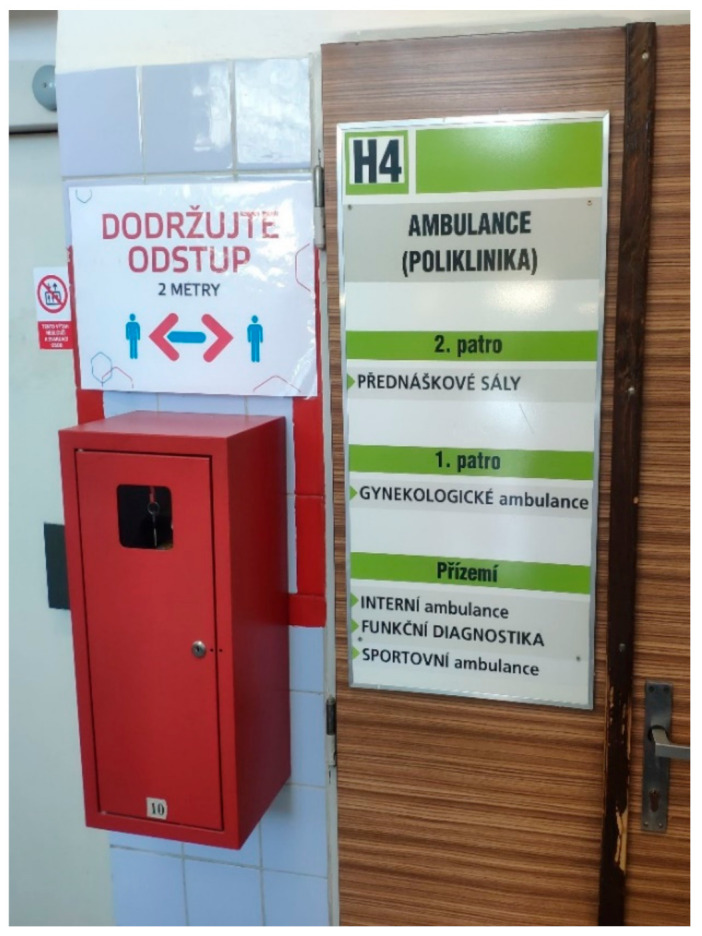
Door signs in the Vítkovice Hospital (10/2020).

**Figure A2:**
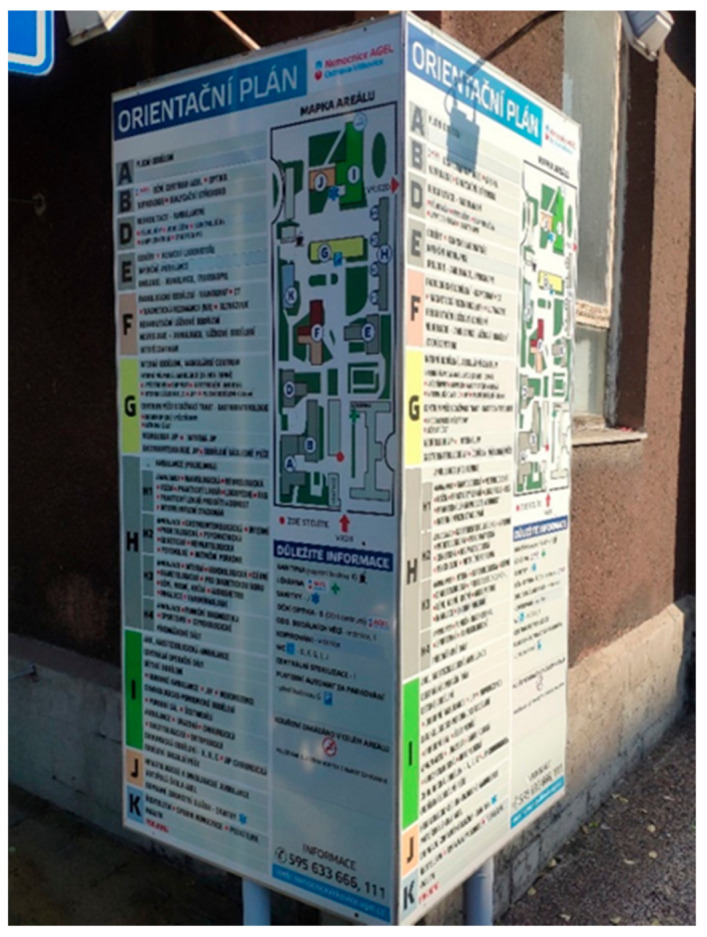
Signs in the Vítkovice Hospital (10/2020). Photo taken by the authors.

The current navigational systems in the area correspond to the common standards in Czech facilities of a similar type (Figure A1 and Figure A2). Upon entering the complex, a map of the whole area is available. Individual buildings in the area are marked with coloured letters. However, there is no link between the letters and the names of individual departments. In the hospital complex, there are signs on various buildings that direct patients within the outdoor complex. These signs are often placed randomly. The surgeries and wards in the interior are marked by a sign at the door, including office hours (in the case of ambulances). The department is marked with a poster on the front door. Before entering the building, in the lifts and on the given floor, there are always orientation signs with the name of the department/ambulance, which are changed on the given floor (floors in the case of the building and the elevator).

There are no maps or graphical representations of individual departments in the buildings. Orientation signs with a list of surgeries/ departments in a given building or designation of departments/ambulances on a given floor are located everywhere. If patients cannot enter some parts of the premises or the buildings themselves, there are signs prohibiting entry. All buildings and departments are fully barrier-free. If a patient wants to plan the trip in advance, information about the location of the department is available on the website

## 4. Data and Methods

We considered several options for data collection and decided to use structured questionnaires in the paper form. The questionnaire aimed to determine if, and to what extent patients perceived the hospital navigation system as inadequate, and to what extent smart technologies should be used to improve wayfinding in the Vítkovice Hospital. However, we did not prefer technological solutions in the questionnaire in order not to influence the respondents and to get undistorted information about their willingness to use them. Design of the questionnaires was based on the focus groups (inspired by the research of Brown et al. [70], including the doctors and other medical and technical staff.

In the first step, a pilot survey of 100 respondents was conducted to check the adequacy and comprehensibility of the questionnaires. Several questions were adapted to increase their comprehensibility for the target group. In June 2019, we distributed 4000 questionnaires to the selected departments of Vítkovice Hospital. Questionnaires were distributed according to the mean weakly number of patients in the departments. The survey took place between 26th June and 3rd July to collect the answers from all departments during the working week, and 920 filled questionnaires were collected. Respondents completed 13 questions focusing on their experience with wayfinding in the hospital, willingness to use the smartphone application for navigation, other preferred types of navigation, as well as demographic characteristics, such as gender, age, and education (Table 1). Several respondents did not answer some questions correctly, or did not answer them at all. For example, the question 11 on gender was answered by 921 respondents, the questions 12 and 13 on age and education were answered by 924 respondents. Number of valid responses (*N*) thus slightly differed among various questions.

### Source: The Authors

Variables 6, 7, 9, and 10, capturing navigational needs of the patients/visitors and their willingness to use the smartphone application for navigation in the hospital, entered the statistical tests as dependent variables. We tested the effects of the explanatory variables 1–5, 8, and 9 using the chi2 tests. We also tested the interactions of explanatory variables: age and gender (men 18–39; men 40+; women 18–39; women 40+), education and gender (men with elementary/secondary/tertiary education; women with elementary/secondary/tertiary education), and finally, education and age (elementary/secondary/tertiary education 0–39; elementary/secondary/tertiary education 40+). Besides, we excluded all respondents who visited the hospital department regularly, and analyzed only the answers of the newcomers and respondents who visited the hospital department after a long time. The reason was to control the effects of the experience—several respondents might not demand any change in the navigational system, because they are used to the existing (while inefficient) mode of wayfinding.

## 5. Results

Let us start with basic descriptive statistics of the survey (Table 2). Respondents who were either newcomers or people who visited the hospital after a long time amounted to 44.1%. Our sample was dominated by the age group 60+, visiting the hospital at least sometimes, or even regularly. These respondents could view the issues of wayfinding differently and might have been rather reluctant to accept possible changes and improvement in the navigational system. Therefore, we decided to run two rounds of statistical tests. In the first step, we tested the effects of age, gender, and education in the group of all respondents. In the second step, only newcomers and people visiting the hospital department after a long time were considered.

**Figure 1:**
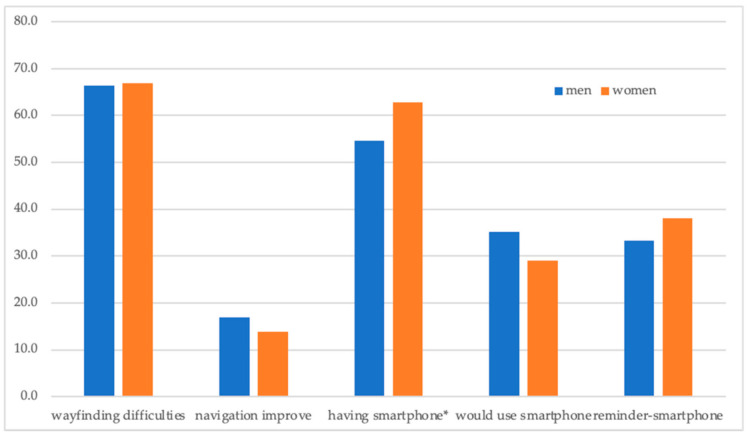
Gender and opinions of the respondents: share of positive answers (%). Source: The Authors. Note: variables significantly related to the gender are marked: * *p* < 0.05.

Perhaps the most important information is that roughly two-thirds of respondents agreed that newcomers might face serious difficulties in wayfinding (Figure 1). This contrasts with a modest (16.9% men; 13.9% women) share of respondents who would appreciate the improvement in the navigational system of the hospital. More than half of men and almost two-thirds of women had a smartphone, but only 35.2% of men and 29% of women would use a smartphone application for navigation in the hospital.

**Figure 2:**
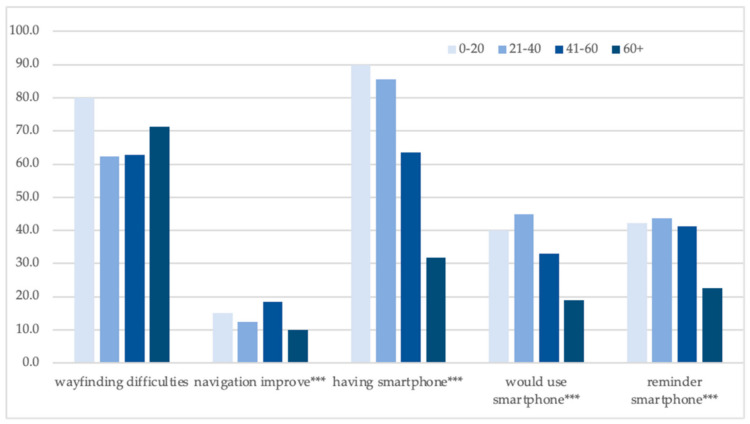
Age and opinions of the respondents: share of positive answers (%). Source: The Authors. Note: variables significantly related to the age are marked: * *p* < 0.05; ** *p* < 0.01; *** *p* < 0.001.

Perception of wayfinding difficulties shows relatively small differences among the age groups (Figure 2). A high share of positive answers in the age group of 0–20 years can be distorted by their small number. Navigation improvements were required mostly by the age group of 41–60. A share of positive answers on the ownership/willingness to use a smartphone for navigation and reminders sent by SMS were negatively correlated to the age—respondents aged 60+ were generally less willing to use mobile applications.

**Figure 3:**
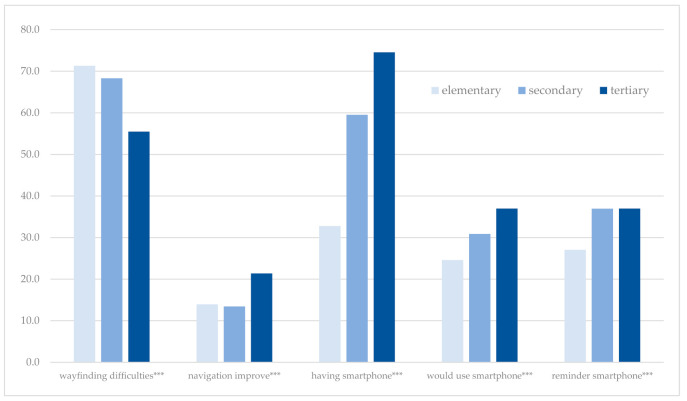
Education and opinions of the respondents: share of positive answers (%). Source: The Authors. Note: variables significantly related to the education are marked: * *p* < 0.05; ** *p* < 0.01; *** *p* < 0.001.

We also found a positive association between education and demand for navigation improvements, either through mobile applications or other ways (Figure 3). Respondents with tertiary education were more interested in navigation improvements and more willing to use mobile navigation applications. On the other hand, wayfinding difficulties for newcomers were more often found by respondents with elementary education.

**Figure 4:**
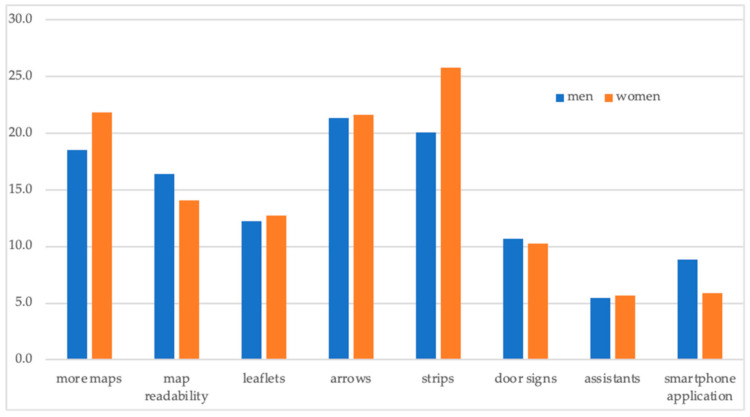
Navigational preferences of the respondents: share of positive answers (%). Source: The Authors.

If we compare particular ways of navigation (Figure 4), a smartphone application was among the least popular solutions, especially among women. On the other hand, when we asked our respondents what should be improved, they mostly agreed on colour strips on the floor (mostly women), more signs/arrows, and more maps in the hospital. There were no major differences between men and women in their navigational preferences.

**Figure 5:**
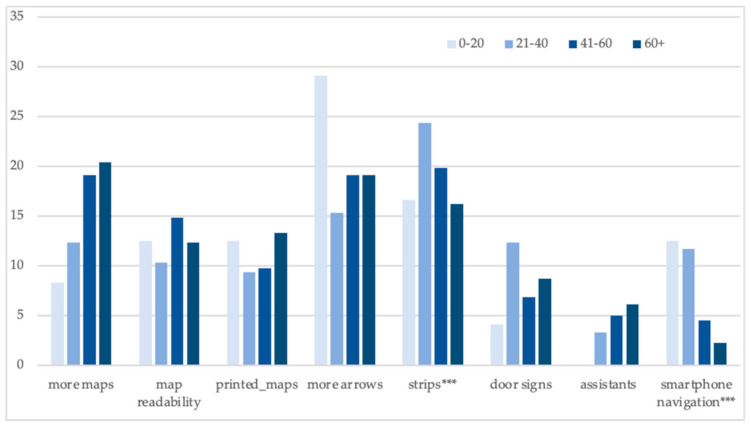
Age and navigational preferences: share of positive answers (%). Source: The Authors. Note: variables significantly related to the age are marked: * *p* < 0.05; ** *p* < 0.01; *** *p* < 0.001.

Navigational needs and preferences varied more among various age groups than between men and women (Figure 5). We found a positive association between age and demand for maps and verbal instruction from the assistants/hospital staff. Younger respondents preferred smartphone navigation applications considerably more than respondents aged 40+.

**Figure 6:**
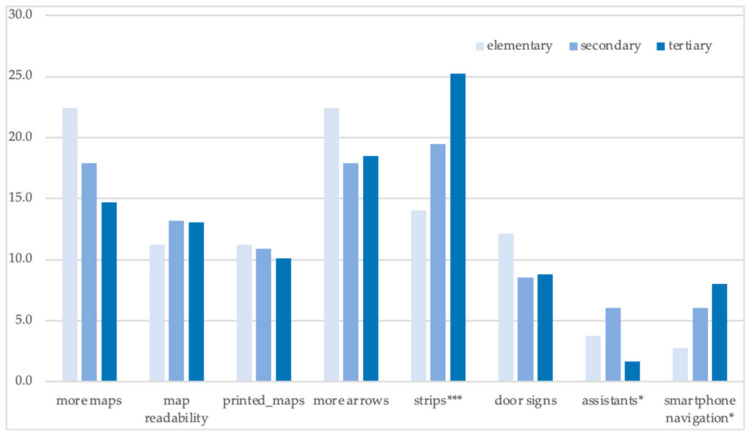
Education and navigational preferences of the respondents: share of positive answers (%). Source: The Authors. Note: variables significantly related to the education are marked: * *p* < 0.05; ** *p* < 0.01; *** *p* < 0.001.

There were no consistent effects on the educational level (Figure 6). Respondents with elementary education requested more maps, arrows, and door signs. Surprisingly, people with tertiary education appreciated colour strips and—as expected—smartphone navigation applications.

Now, we turn to the regression models capturing the effects of age, gender, and education on wayfinding in general and the willingness to use a smartphone application for navigation in the hospital.

### 5.1. Age

We found a highly significant (*p* < 0.01), but ambiguous effect of age on navigational preferences (Table 3). Only 25.2% of the age 60+ respondents would appreciate an improvement in the navigational system of the hospital. The only group demanding an improvement in the navigation were respondents aged between 41 and 60 years. We found no association between the age and the opinion on the difficulties with wayfinding for the newcomers. When we asked the respondents which navigational strategy they used to find their department, younger respondents (up to age 60) either used maps, webpages of the hospital, or asked the staff more often than those aged 60+. On the other hand, those aged 60+ asked more often at the reception. No significant differences between the age groups were found in their usage of arrows, instructions from the doctors or nurses, information on the prescription, or asking other patients. Answers on the questions concerning the smartphones (having, would use for navigation, would appreciate an SMS reminder) were related negatively to the age—older respondents preferred mobile navigation less than those who were younger.

### 5.2. Source: The Authors

Not surprisingly, we found a strong negative relationship between age and usage of smartphones. Of those aged 60+, 61.4% reported that they had/used no smartphone. Correspondingly, with increasing age, the share of respondents willing to use a smartphone for navigation in the hospital rapidly fell (*p* < 0.01). We also found a relatively weak association between the age and preferred type of navigation in the hospital. There were no significant differences between the age groups in their demand for more/better maps, arrows, signs, and help from staff. Respondents between 41 and 60 years would appreciate colour strips, while those 60+ would not. The only group that demanded better door signs were people between ages 21 and 40. Smartphone navigation and the reminders sent to their smartphones would be appreciated only by the people younger than 40 years (*p* < 0.01).

### 5.3. Gender

Gender was another variable partially associated with wayfinding. Men and women differed neither in their frequency of visits to the hospital/department, nor in their perception of the wayfinding difficulties for the newcomers. Women use smartphones more often than men, but at the same time, women are less willing to use smartphone applications for navigation in the hospital. There are no significant associations between gender and demand for maps, arrows, signs, door signs, and assistants showing the way. On the other hand, women would appreciate colour strips on the floor more than men. There were minor differences in the way that men and women found their particular department at times of the data collection. Men used maps more often, while women relied more on the hospital webpages. While women demanded an improvement of the navigation system more than men, the results were not statistically significant.

### 5.4. Education

Less-educated respondents (with elementary education) visit the hospital more often than respondents with a university education, which might skew the results. Educated respondents are more sensitive to the navigational problems of the newcomers. There are only minor and predictable differences between people with elementary/secondary/tertiary education in their wayfinding strategies. Respondents with university education preferred the smartphone applications more as well as, surprisingly, colour strips, while those with secondary education appreciated help from the staff. Considering the question of how they got to their particular department, respondents with university education used maps and webpages more often, while people with elementary education were more willing to ask at the reception. We also found a positive association between education and support for the improvement of the navigational system in the hospital. There was a strong positive association between education and ownership of a smartphone. The same holds for the willingness to use the smartphone application for navigation of the hospital and SMS reminders from the hospital.

In the second step, we tested the effects of the interactions between age, gender, and education. While we found no effect of the interaction between age and gender on wayfinding difficulties of newcomers, a relationship with the demand for improvement of the navigational system in the hospital was significant. Men aged 40+ were the only group showing above-average interest for the improvement in navigation. Only age (rather than its interaction with gender) affects current usage and willingness to use a smartphone and preference of various types of navigation—younger respondents favoured mobile applications, colour strips, and door signs, rather than age 40+ respondents.

Correspondingly, education affects wayfinding strategies more significantly than gender. Several differences between respondents with elementary, secondary, and tertiary education were noted. On the other hand, gender differences within the same educational level are relatively modest. Women with tertiary education used hospital webpages more than any other demographic group. Mobile navigation apps were demanded by men with secondary and tertiary education, and by women with only tertiary education. Maps were used for navigation more by men with secondary and tertiary education and by women with tertiary education. In almost all significant relationships between the education × gender and wayfinding strategies, we observed a higher preference of respondents with secondary and/or tertiary education.

Considering the interaction between education and age, the latter variable is a more important predictor of the willingness to use mobile applications. However, while age 40+ respondents would use mobile applications less than their younger counterparts, tertiary education increases willingness to use mobile applications in the age 40+ group. Surprisingly, the only group that appreciated an improvement in the hospital navigation system were respondents aged 40+ with tertiary education. If we look at navigational preference, respondents aged 40+ with elementary education used maps significantly less than other groups.

Finally, we also tested the effects of demographic variables in the sample of newcomers and respondents who visited the hospital/department a long time before the survey. Considering the association between gender and navigational preferences, we found only one significant difference—men were more willing to use the smartphone application than women. People with university education were more eager to use the smartphone application as well as the colour strips, and other relationships were either inconclusive or insignificant.

## 6. Discussion

Let us start with a discussion of age, gender, and education as factors moderating the willingness to use new technologies [71], more particularly using smartphone navigation in the hospital. If we return to the digital divide model [60] predicting that women, and younger and less educated people are less prone to adopt new technologies, we found empirical support only for the effects of age and education. Older and less educated respondents showed significantly lower interest in smartphone navigation and, correspondingly, also lower usage of smartphones. No significant differences between men and women in their opinion on the usefulness of the smart navigation system were found.

It is not clear whether a negative association between age and willingness to use a mobile app was caused by difficulties in learning of new technologies (as suggested by Morris et al. [72]), or if older respondents relied more on habit and were used to the current hospital navigation system. These explanations might go hand in hand. Venkatesh et al. [71] argued that gender differences in the adoption of new technologies increase with age. According to the authors, older men with more usage experience relied on their habits more than women and may be more reluctant to adopt new technology. Younger women with less usage experience are more sensitive to innovative cues and rely significantly less on habit. Younger men, on the other hand, are most likely to adopt completely new technology, because they put lesser emphasis on the facilitating conditions (technical support; price, etc.) than women (especially older). Because a mobile navigation app cannot be nowadays considered a completely new technology, younger women should be the most, and older men, the least sensitive demographic group to this change.

Hypotheses 3 and 4 expected gender differences in the opinion that the newcomers face difficulties in orientation in the hospital and the demand for a new navigational system. We did not find sufficient empirical support to prove them. There were no major differences between men and women in their perception of wayfinding difficulties for newcomers. Women would appreciate an improvement in the navigation system more than men (in line with the expectations of Venkatesh et al. [71]), but the results were not significant. Therefore, higher spatial anxiety of women [41] did not translate into major gender differences in navigation requirements. Besides, experience with the hospital showed a surprisingly weak effect on navigational preferences.

Age and education were more pronounced in the navigational preferences of the respondents than gender. Although women demanded an improvement in navigation more than men, this effect was mediated by age. Men aged 40+ (but also men aged 60+) demanded an improvement in the navigational system more than any other demographic group. This contrasts with an assumption of Venkatesh et al. [71] that older men rely on habit in using technologies more than women. More educated respondents preferred improvements in navigation and using mobile applications more than respondents with elementary education: this effect was slightly stronger for men than women.

We confirmed no significant gender differences in the preference of navigational cues. According to the theory [38], men use more absolute directions and Euclidean distances, while women rely more on a relative frame (e.g., left, right). However, Anacta and Schwering [14] found that both genders performed (in terms of wayfinding) better when the route instructions were given in relative frame. Liao and Dong [43] argued that despite a preference of absolute directions, men can also use landmarks to find a destination. Real wayfinding strategies in large complex buildings might not differ so much between men and women. The only significant difference we found was the way in which the respondents found the hospital department—men used maps more often, while women checked the hospital webpages. Therefore, both genders can appreciate similar navigational cues, such as signs or landmarks. We also did not prove that women preferred verbal instructions more than men, as suggested by Devlin and Bernstein [58] or Wilkening and Fabrikant [59].

## 7. Suggested Solutions

The proposed solution can be implemented in large complexes of buildings, such as shopping malls, airports, or university campuses. In the first phase, it is necessary to map the area, which consists of buildings. This is followed by detailed mapping of individual buildings, including all floors, rooms, stairs, elevators, and ramps. Subsequently, it is necessary to define the so-called points of interest in individual buildings. This means points where the visitor to the complex will find the service. In the case of hospitals, these are individual ambulances, examination rooms, inpatient departments, and so forth. If we have mapped important points in buildings, it is necessary to define individual routes between these points. These are routes with predefined attributes. Attributes define various restrictions of given routes, as well as time possibilities with different types of movement. Especially in the case of a hospital, visitors are variously restricted in their movement, such as by using a wheelchair. The use of these means of transfer, therefore, places different demands on the route, and it is not possible to use a staircase, but only a lift.

By defining individual points of interest and individual routes between them, we get the data structure of the graph, where individual points of interest are nodes and routes of the edge of the graph. This data will be uploaded to the PostGIS geodatabase, where the shortest path (such as Dijkstra’s algorithm) will be searched using the pgRouting network analysis algorithms (pgrouting.org).

GIS resources allow you to have all available data (maps, points of interest, routes) on a web server as a web application that can be accessed through any browser. This web application allows, for example, visitors to find the shortest route according to the input. It can use any mobile device with a web browser. GPS sensors can be used to determine the position for movement between individual buildings and, to a limited extent, in buildings. Because the possibilities of the GPS signal are limited in buildings, there will be a QR code in clearly visible places to specify the visitor’s location. The client application for navigation in the building will also allow visual (or voice) information about the direction to the destination. The point is that even users with various disabilities can use this system. Emphasis will be placed on the simplicity and intuitiveness of control so that everyone can control the application.

A service application will be used to update the data in the server part of the application, which will allow system administrators to enter data that have changed. In the case of a hospital, this may be a transfer of the relevant ambulance, either temporary with a time interval or a permanent transfer. Similarly, office hours can be updated according to the situation. These changes take place online in real-time, so individual users immediately use up-to-date data for navigation in buildings.

For visitors without mobile devices, information panels will be available with the option of finding the shortest route to the specified point of interest. These information panels can be in the form of information kiosks at the entrances to the premises or at the intersections of corridors. These information panels and QR codes for positioning create a network of information navigation points in buildings, according to which autonomous devices (such as an autonomous wheelchair) can also be navigated in buildings in the future.

## 8. Conclusions

In this paper, we aimed to identify current navigational strategies, major barriers and obstacles in wayfinding of the hospital visitors, and their willingness to use a mobile navigation application. Our empirical analysis was based on a survey of 928 respondents in the Vítkovice Hospital in Ostrava, Czechia. Roughly two-thirds of respondents answered that newcomers may face serious difficulties in wayfinding. However, only less than a third of them would appreciate the improvement in the hospital navigational system: respondents between 41–60 years old, more than 0–39 years old, and 60 years of age or older respondents, women slightly more than men, and educated respondents much more than people with elementary education. It is a question of whether hospital visitors were not interested in navigation improvement, or if they were not motivated enough to complete the questionnaire thoughtfully.

In the empirical part of the paper, we tested the effects of age, gender, and education on wayfinding strategies and needs. Surprisingly, there were no major gender differences in navigational preferences. Contrasts in wayfinding postulated by the theory (women—egocentric vs. men—allocentric navigation strategies) were almost not reflected in actual navigational preferences. Apart from the colour strips on the floor that were preferred more by women, there were no major gender differences in the preference of various cues, such as maps, signs, or written or verbal instructions. A smartphone application was among the least popular solutions, especially for older respondents with elementary education.

Age was the most important predictor of wayfinding. Respondents aged 60+ were characterised by a lower interest in changes of the navigational system and low willingness to use mobile applications for navigation; people between 41 years and 60 years old were the biggest supporters of changes. Correspondingly, demand for improvement of navigation (including a mobile application) was positively correlated with educational level.

To sum up, respondents who may face the biggest difficulties with wayfinding (elderly, elementary education) are mostly not interested in improvements in the navigational system. The hospital has to consider their navigation needs and requirements without having sufficient feedback from them. We suggest that the research focused on patients and hospital visitors should be complemented by observation of navigational strategies of the patients and hospital visitors, and also by a survey among the hospital staff. Doctors, nurses, and administrative and technical workers might help to identify the most problematic places of where the visitors get lost and also where/why they ask hospital staff for help.

## Figures and Tables

**Table 1:** Variables employed in the statistical test.

Variable Number	Name of the Variable	Description
1	Experience-hospital	You visit this hospital: 1 = for the first time; 2 = not for the first time, but after a long time; 3 = sometimes; 4 = regularly.
2	Experience-department	You visit this department: 1 = for the first time; 2 = not for the first time, but after a long time; 3 = sometimes; 4 = regularly.
3	Wayfinding	Is it easy to find the place for the newcomers? 1 = easy; 2 = some difficulties; 3 = difficult.
4	Finding-department	How did you find your department? 1 = knew it before; 2 = looked at the map of the hospital; 3 = used signs; 4 = instructions from the sending doctor; 5 = instruction from the sending nurse; 6 = it was written on the prescription; 7 = asked the staff; 8 = asked another patient; 9 = asked the doorman; 10 = found on the webpage; 11. other.
5	Arrival	When did you arrive to the department? 1 = I was not ordered on the exact time; 2 = ahead of time/on time; 3 = lately due to getting lost in the hospital; 4 = lately-another reason.
6	Navigation-improve	Navigation improvement is needed: 1 = strongly agree; 2 = agree; 3 = disagree; 4 = don’t know.
7	Navigation-prefer	Which type of navigation would you prefer? 1 = more maps; 2 = map readability; 3 = printed maps; 4 = more arrows; 5 = strips; 6 = door signs; 7 = assistents; 8 = smartphone navigation; 9 = other.
8	Having-smartphone	1 = I am using and having in now; 2 = using, but not having now; 3 = using, but not in the hospital; 4 = not using.
9	Using-smartphone	Would you use a smartphone for navigation in the hospital? 1 = definitely yes; 2 = only in case of getting lost; 3 = rather not; 4 = no.
10	Reminder-smartphone	Would you like to get a reminder of the physical examination in the hospital on your smartphone? 1 = definitely yes; 2 = I would not mind; 3 = rather not; 4 = no.
11	Gender	0 = male; 1 = female
12	Age	1 = 0–20 years; 2 = 21–40; 3 = 41–60; 4 = more than 60 years
13	Education	1 = no/elementary; 2 = secondary; 3 = tertiary

Note: variable 7: more maps = more orientation maps in the hospital complex; maps readability = improving the clarity and readability of the maps in the hospital complex; printed maps = printed plans of the hospital provided to all visitors at the entrance; more arrows = more orientation arrows and signs in the hospital complex; stripes = guiding coloured stripes on the ground; door signs = better and more illustrative door signs; assistants = staff helping with the wayfinding in the hospital; smartphone navigation = mobile application for navigation in the hospital complex and buildings.

**Table 2:** Structure of the respondents: descriptive statistics.

	Number	Share (%)
Gender	–	–
men	384	41.7
women	537	58.3
–	–	–
Age	–	–
<20	20	2.2
21–40	223	24.1
41–60	358	38.7
60	323	35.0
–	–	–
Education	–	–
elementary	124	13.4
secondary	626	67.7
tertiary	174	18.8
–	–	–
Experience with the hospital department	–	–
newcomers	274	29.7
coming after a long time	133	14.4
visiting sometimes	227	24.6
regular visitors	288	31.2

Source: own survey.

**Table 3:** Effects of the gender, age, and education on wayfinding (selected tests).

Tested Variables	*N*	χ^2^	*p*
Gender X Wayfinding difficulties	911	0.482	0.786
Gender X Finding department-map	921	3.498	0.064
Gender X Finding department-arrows	921	1.357	0.262
Gender X Finding department-asked-staff	921	0.162	0.745
Gender X Finding department-asked-patient	921	0.618	0.507
Gender X Finding department-asked-reception	921	0.560	0.469
**Gender X Finding department-webpage**	**921**	**4.570**	**0.033**
Gender X Navigation-improve	913	6.047	0.109
Gender X Navigation prefer-more-maps	921	1.189	0.281
Gender X Navigation prefer-map-readability	921	1.219	0.302
Gender X Navigation prefer-more-arrows	921	0.002	0.964
Gender X Navigation prefer-strips	921	3.503	0.068
Gender X Navigation prefer-assistants	921	0.006	0.939
Gender X Navigation prefer-smart-app	921	3.241	0.089
**Gender X Navigation-have-smart**	**913**	**8.299**	**0.040**
Gender X Navigation-using smart	913	6.230	0.097
Gender X Reminder-smartphone	911	3.633	0.304
Age X Wayfinding difficulties	914	9.792	0.134
**Age X Finding department-map**	**924**	**9.008**	**0.029**
Age X Finding department-arrows	924	1.456	0.693
Age X Finding department-asked-staff	924	7.046	0.070
Age X Finding department-asked-patient	924	1.153	0.764
**Age X Finding department-asked-reception**	**924**	**13.725**	**0.003**
**Age X Finding department-webpage**	**924**	**12.872**	**0.005**
**Age X Navigation-improve**	**916**	**32.002**	**<0.001**
Age X Navigation prefer-more-maps	924	5.566	0.135
Age X Navigation prefer-map-readability	924	5.693	0.128
Age X Navigation prefer-more-arrows	924	5.084	0.166
**Age X Navigation prefer-stripes**	**924**	**22.569**	**<0.001**
Age X Navigation prefer-assistants	924	1.977	0.577
**Age X Navigation prefer-smart-app**	**924**	**40.365**	**<0.001**
**Age X Navigation-have-smart**	**916**	**207.513**	**<0.001**
**Age X Navigation-using smart**	**916**	**147.558**	**<0.001**
**Age X Reminder-smartphone**	**914**	**100.262**	**<0.001**
**Education X Wayfinding difficulties**	**914**	**31.122**	**<0.001**
**Education X Finding department-map**	**924**	**6.664**	**0.036**
Education X Finding department-arrows	924	3.122	0.210
Education X Finding department-asked-staff	924	2.988	0.224
Education X Finding department-asked-patient	924	0.016	0.992
**Education X Finding department-asked-reception**	**924**	**8.193**	**0.017**
**Education X Finding department-webpage**	**924**	**10.596**	**0.005**
**Education X Navigation-improve**	**916**	**31.044**	**<0.001**
Education X Navigation prefer-more-maps	924	0.079	0.961
Education X Navigation prefer-map-readability	924	3.853	0.146
Education X Navigation prefer-more-arrows	924	2.204	0.332
**Education X Navigation prefer-strips**	**924**	**21.384**	**<0.001**
**Education X Navigation prefer-assistants**	**924**	**6.898**	**0.032**
**Education X Navigation prefer-smart-app**	**924**	**8.081**	**0.018**
**Education X Navigation-have-smart**	**916**	**57.054**	**<0.001**
**Education X Navigation-using smart**	**916**	**27.779**	**<0.001**
**Education X Reminder-smartphone** **Interactions**	**914**	**52.947**	**<0.001**
Age × Gender X Wayfinding difficulties	910	1.497	0.960
**Age × Gender X Navigation-improve**	**912**	**18.105**	**0.034**
Education × Gender X prefer-smart-app	920	10.576	0.056
**Education × Gender X would-use-smartphone**	**912**	**36.286**	**0.002**
**Education × Age X would-use-smartphone**	**915**	**119.447**	**0.000**
**Education × Age X Navigation-improve**	**915**	**50.734**	**0.000**

Note: *N* = number of valid answers; χ^2^ = results of chi2 test; *p* = significance (*p*-value). Significant results (*p* < 0.05) are marked bold. X = dependence between two variables.

## Data Availability

Data is contained within the article.
